# The impact of predictability on dual-task performance and implications for resource-sharing accounts

**DOI:** 10.1186/s41235-020-00267-w

**Published:** 2021-01-04

**Authors:** Laura Broeker, Harald Ewolds, Rita F. de Oliveira, Stefan Künzell, Markus Raab

**Affiliations:** 1 Institute of Psychology, German Sport University Cologne, Am Sportpark Müngersdorf 6, 50933 Cologne, Germany; 2 Institute of Sports Science, Augsburg University, Universitätsstraße 3, 86135 Augsburg, Germany; 3 School of Applied Sciences, London South Bank University, 103 Borough Road, London, SE1 0AA UK

**Keywords:** Multitasking, Dual task, Predictability, Task integration, Tracking

## Abstract

The aim of this study was to examine the impact of predictability on dual-task performance by systematically manipulating predictability in either one of two tasks, as well as between tasks. According to capacity-sharing accounts of multitasking, assuming a general pool of resources two tasks can draw upon, predictability should reduce the need for resources and allow more resources to be used by the other task. However, it is currently not well understood what drives resource-allocation policy in dual tasks and which resource allocation policies participants pursue. We used a continuous tracking task together with an audiomotor task and manipulated advance visual information about the tracking path in the first experiment and a sound sequence in the second experiments (2a/b). Results show that performance predominantly improved in the predictable task but not in the unpredictable task, suggesting that participants did not invest more resources into the unpredictable task. One possible explanation was that the re-investment of resources into another task requires some relationship between the tasks. Therefore, in the third experiment, we covaried the two tasks by having sounds 250 ms before turning points in the tracking curve. This enabled participants to improve performance in both tasks, suggesting that resources were shared better between tasks.

According to capacity-sharing accounts, people can flexibly allocate generic processing resources to different competing tasks and stages of processing, which allows concurrent dual tasking (cf. Koch et al. 2018, regarding a flexibility perspective in multitasking; Meyer and Kieras 1997). Limitations in dual-task processing may however occur because the total amount of utilizable resources is limited and may be depleted once different stimulus and response modalities draw on this pool of central attentional resources [Kahneman 1973; Posner and Petersen 1990; Tombu and Jolicœur 2003; but see Wickens (2002, 2008), for a modality-specific resources account].

With a limited pool of processing resources, critical aspects to successful dual tasking would thus be the reduction of required resources and an effective resource allocation policy. In this study, we have examined the impact of predictability on dual-task performance and its potential implications for a reduction in resource needs and resource allocation policy. Considering that prediction is a permanently ongoing process of the human perceptual, cognitive and motor system (Bubic et al. 2010; de Oliveira et al. 2014) and single-task (ST) studies have shown that predictable tasks are processed more efficiently and require fewer attentional resources [as indicated by decreased cortical activity, see Eagleman et al. (2009], we expected predictability to also have an impact on resource utilization in dual tasks. This view has also been supported by Wahn and König (2017). They claimed that the degree to which a stimulus in the environment can be predicted influences the allocation of attentional resources, and that one future direction for research is to “investigate the extent to which attentional resource limitations can be circumvented by varying the predictability of the presented stimuli” (p. 91). In continuous tasks, predictability may take the form of visual information about the route ahead enabling participants to plan action required in a few milliseconds (de Oliveira et al. 2014). Predictability thus leads to an optimal configuration of the sensory system prior to stimulus onset, which facilitates processing of environmental input (Fougnie et al. 2018; Król and Król 2017). For this reason, predictability may be of particular importance in dual-task (DT) situations; if predictable tasks require fewer resources, then there should be residual resources available for the other task. For settings where the *primary* task is predictable, this process has also been termed the trickle-down effect of predictability (Król and Król 2017). None of the capacity-sharing accounts do however provide hypotheses about the utilization of residual resources. This might be due to the fact that testing the utilization of resources and residual resources is difficult as the metaphorical construct “resource” is not directly measurable or quantifiable [for a critical evaluation of dual-task theories see Hommel (2020)]. In the literature however, any reduction in costs (either comparing single against dual tasks, or comparing two different dual tasks) and improvements on the dependent variable have been accepted as a proxy for reduced resources (e.g., Fougnie et al. 2018; Gopher et al. 1982; Wahn and König 2015). In addition, it seems advisable to not only look at performance improvements in the primary tasks or dual-task costs (difference between single- and dual-task performance), but to also report performance, and potentially changes, in the secondary task. A closer look at secondary task performance might give an indication of how resources are allocated.

We hypothesize that the human system draws on one general pool of resources [Kahneman 1973; Tombu and Jolicœur 2003; for an opposing view see Wickens (2008), as well as the discussion below], and that a predictable primary task frees resources that can be used for a secondary task. This allocation policy should result in improved performance in both tasks. On the contrary, if predictability improves performance in only one (the predictable) task, this would be in line with the previously suggested economic processing mode where humans aim to reduce, not reinvest resources (see also Navon and Gopher 1979; Plessow et al. 2012). In the literature, there is evidence for both secondary tasks benefiting from a predictable primary task (Cutanda et al. 2015; Töllner et al. 2012) and for improved performance in the predictable task but not in the secondary task (Corr 2003; Ewolds et al. 2017). For instance, Cutanda et al. (2015) showed that when participants concurrently performed an irregular vs. rhythmic auditory response task with an N-back memory task, they responded faster after regular rhythms compared to irregular rhythms, and this was regardless of memory load. By contrast, Ewolds et al. (2017) used a tracking task which became predictable through learning the track over several days. They showed that performance in a tracking task improved, yet reaction times to the auditory secondary task did not differ between the reactions needed during the learnt versus random tracking segments. Taken together, there is both limited and conflicting empirical evidence regarding the benefits of predictability in the primary task on the secondary task. On the other hand, there is empirical evidence that resource allocation policy can be influenced, and consequently that resources can be unevenly distributed among tasks. For instance, instructing participants to put more emphasis on one vs. the other task (Lehle and Hubner 2009; Tsang 2006), different perceptions of potential outcome value and the saliency of tasks (Schmidt and Dolis 2009; Wickens et al. 2003, 2015; Wickens and Colcombe 2007), or distractions during dual-task execution (Strayer and Drews 2007) can impact resource allocation policy. However, these studies do not report what implications such an allocation policy might have for the other task which is why further attention should be given to potential drivers of resource reduction and allocation in order to optimize dual-task behavior (Salvucci and Taatgen 2008; Tombu and Jolicœur 2003).

In this study, we have taken a systematic approach, manipulating predictability in the first task, in the second task, and in both tasks to examine the impact of predictability on dual-task performance and the implications for resource reduction and resource allocation policies.

We used a continuous visuomotor tracking paradigm together with a discrete auditory reaction time task, because it has been shown that this combination of tasks reliably leads to dual-task costs (Ewolds et al. 2017; Fougnie et al. 2018; Lang et al. 2013). More importantly, tracking tasks allow the measure of temporal-spatial variables (i.e., velocity) which give insight into performance changes as soon as another task intervenes. If velocity increases or decreases once participants respond to the auditory task, this indicates that resources are taken away from tracking and we can make inferences about the resource allocation policy. Predictability was manipulated by displaying parts of the tracking path (Experiment 1) and sequencing sounds in the auditory task (Experiment 2a/b). In Experiment 3, we covaried both tasks by playing target sounds 250 ms before the inflection points of the tracking curve and as such the auditory task could be used to predict changes in the tracking task. The covariation created a meaningful relation between tasks, serving as an incentive for participants to reinvest resources into this task or even integrate the tasks into one (Ewolds et al. 2020; Schmidtke and Heuer 1997).

## Experiment 1

Considering that predictability is provided by information in the environment or prior knowledge of a person (Gentsch et al. 2016; Körding and Wolpert 2006; Wolpert et al. 2003), the first experiment manipulated predictability in the tracking environment by providing participants with advance visual information about the tracking path. The continuous task provides a suitable paradigm to examine the hypothesized processes of resource allocation because it allows for flexible scheduling, in contrast to using two discrete tasks. This gives insights into allocation policies at the moment an interfering secondary stimulus occurs. In addition, the information about the tracking path allows feedforward control which can correct positional errors, delays between target and controller, or jerkier trajectories (Engel and Soechting 2000; Hill and Raab 2005; Lange 2013; Scott 2012; Weir et al. 1989; Wolpert et al. 2011). With fewer resources needed in one predictable task there should be residual resources available that can be used for another task. A DT tracking study by Eberts (1987) already showed that participants receiving visual information on both sides of a moving target improve DT tracking performance, but as no reaction times (RTs) for verbal secondary-task responses were reported, a look into the performance on the secondary task is required in order to make inferences about potential resource allocation.

### Methods

#### Participants

In total, 38 participants were recruited on a university campus, via a mailing list or through a participant data bank. Three participants were identified as outliers and were excluded from the analysis, yielding a final sample of 35 participants (22 males and 13 females; aged between 19 and 30 years, *M* = 21.80 years, SD = 2.56). An a priori G*Power (version 3.1.9.2) analysis revealed a required sample size of 32 participants for a test power of 0.80 (effect size *f* = 0.25 for 2 groups (ST vs. DT) and 5 conditions (predictability), *α* = 0.005 corrected for alpha-error accumulation, 1 − *β* = 0.80, *r* = 0.5).

Participants in this and the following experiments had self-reported normal or corrected-to-normal vision, normal hearing ability, and no musculoskeletal or neurological disorders. Participants gave written informed consent prior to the experiment and received a small remuneration for taking part. The experiments were approved by the local ethics committee and conformed to the principles of the Declaration of Helsinki 2013.

#### Setup

**Fig. 1:**
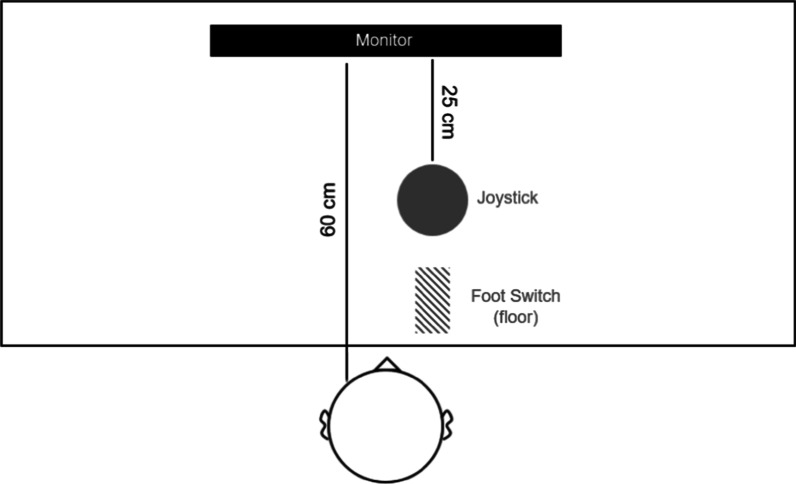
Illustration of the experimental setup

Participants were seated in a dimly lit room at a viewing distance of 60 cm from a 24-in computer screen (144 Hz, 1920 × 1080 pixel resolution). The tracking software ran on a Windows 10, 64-bit system with a GTX750 graphics card. A spring-loaded joystick was fixed to the table 30 cm from the screen (SpeedLink Dark Tornado, max. sampling rate 60 Hz), and the pedal was fixed to the floor under each participant’s self-reported dominant foot (f-pro USB foot switch, 9 × 5 cm; Fig. 1). Participants wore headphones (Sennheiser HD 65TV). The experimenter sat out of view, behind an opaque divider to monitor compliance with the task.

#### Tasks and display

##### Visuomotor tracking task

Participants performed a two-dimensional pursuit-tracking task with a joystick (adapted from Wulf and Schmidt 1997) while concurrently reacting to tones by pedal press. Participants operated the joystick with their self-reported dominant hand and controlled a white cursor cross to track a red target square. Unbeknownst to the participants, the cursor cross’s range of motion was limited to the vertical *y* axis, because its motion on the *x*-axis was coupled to target speed. This was implemented to prevent participants from moving the cursor straight to the right edge of the screen to cut trials short. Every tracking path was composed of three different segments (adapted from Pew 1974), each obeying the formula

$$f\left( x \right) = b_{0} + \mathop \sum \limits_{i = 1}^{6} a_{i} \sin \left( {i \times x} \right) + b_{i} \cos \left( {i \times x} \right)$$

with *a*_*i*_ and *b*_*i*_ being randomly generated numbers ranging from − 5 to 5 and *x* being a real number in the range [0; 2π]. As different amplitudes have been shown to lead to differences in performance (Magill 1998), all randomly generated segments were balanced with regard to mean amplitude beforehand (Wickens 1980). This yielded a final set of 41 segments from which the three segments were selected for each trial. Each trial therefore displayed a different path to prevent participants from learning target trajectories (Ewolds et al. 2017; Van Roon et al. 2008). To avoid the anticipation of peaks (Zhou et al. 2009), the red target followed a constant path velocity of 10.5 cm/s, and as a result, trial length varied from 25.6 to 27.9 s depending on the curve’s trajectory (cf., 27 s used in Raab et al. 2013, and 25 s and 35 s used in de Oliveira et al. 2017).

##### Audiomotor task

The second task was an auditory discrimination task with high-pitched and low-pitched tones occurring randomly along the tracking path (1,086 Hz and 217 Hz, 75-ms duration). Participants reacted to the occurrence of high-pitched tones as fast and as accurately as possible while continuously ignoring the low-pitched tones. Both tones were scaled to the same sound intensity with equal loudness contours (Fletcher and Munson 1933). To avoid learning effects, the number of target and distractor sounds per trial varied between 9 and 14 (every 1.9–3.0 s, following Raab et al. 2013), but all participants received the same total number of sounds across the whole experiment. The first tone appeared no earlier than 500 ms after the trial had started, and to guarantee sufficient response time, the last tone was presented at least 500 ms before the trial ended. Because average RTs for auditory discrimination in earlier DT studies were 500–950 ms (e.g., Bherer et al. 2005), we used a minimum gap between two sounds of 1001 ms, and responses were considered valid only when they were given within 800 ms after the target sound was played.

##### Manipulation of predictability

**Fig. 2:**
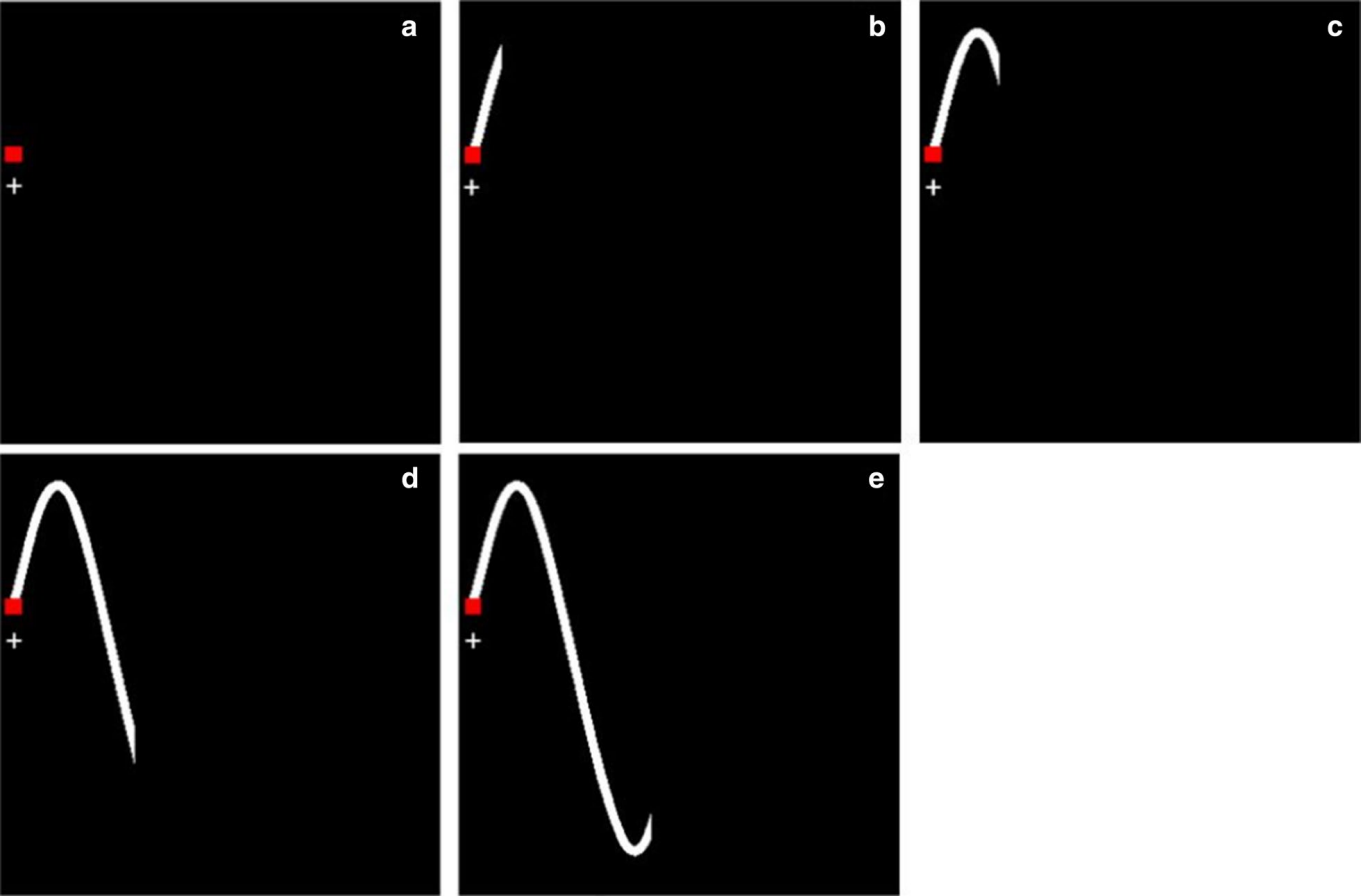
In Experiment 1 participants did not receive any information (**a**; 0 ms) or saw **b** 200 ms, **c** 400 ms, **d** 600 ms, and **e** 800 ms of the tracking path ahead of the red target square. Participants had to follow the red square and its path as accurately as possible by controlling the white cross

The visuomotor tracking task was made predictable by rendering a portion of the tracking path ahead of the target visible (see Fig. 2). The visible path was a white line extending 200 ms (to account for visuomotor delay; e.g., Van Rullen and Thorpe 2001), 400 ms, 600 ms, or 800 ms ahead of the target square (cf., de Oliveira et al. 2014). None of the objects displayed left a trail on the screen. The 0-ms condition represented the unpredictable condition. All five predictability conditions were completed in blocks randomized across participants to avoid training effects (McNeil et al. 2006). High-pitched and low-pitched sounds occurred randomly along the tracking path.

#### Procedure

In the familiarization phase, participants completed two ST tracking trials to become familiar with the joystick, then two ST auditory trials to familiarize themselves with the high- and low-pitched sounds, and finally two DT trials to become familiar with the DT setting. They were told that during the experiment these conditions would appear in random blocks. Participants were instructed to follow the target square as closely as possible, to react to target tones as fast and as accurately as possible, and to put equal emphasis on both tasks. To stimulate motivation, a feedback window informing participants about their tracking performance and RTs popped up after every five trials (McDowd 1986).

In the experimental phase which took approximately 60 min, participants performed 110 trials in total: 50 ST tracking trials (10 × 5 predictability conditions), 10 ST auditory trials, and 50 DT trials (10 × 5 predictability conditions). After completing the experiment, participants answered a questionnaire about their possible use of a specific DT coping strategy. We also asked which predictability condition they felt was the most helpful to improve DT performance by showing five screen shots of the predictability conditions.

#### Data analysis

To measure tracking performance, we calculated the root-mean-square error (RMSE), as a measure of mean deviation from the target tracking path (Wulf and Schmidt 1997; 1 RMSE ≅ 0.56 cm on screen). Performance on the audiomotor task was evaluated by RTs and errors for target sounds. We also measured participants’ absolute velocities. As outlined above, the *target* moved at a constant path velocity meaning the *tracking cross* had the same *x* coordinates as the target. Participants could control upward and downward movement of the tracking cross on the *y*-axis only. Thus, the tracking cross’s velocity was composed of participants’ *y* values and the path’s *x* values and mirrored participants’ speed changes on the *y* axis. Velocities make it possible to investigate changes in tracking behavior at different intervals around the discrete auditory event. We computed four velocity intervals,^1^ one prior to and three after target sound onset: *200 ms before* sound onset until the moment of sound onset; *200 ms after*, which was 75–200 ms after sound onset (given audiomotor delay of 75 ms; Vu and Proctor 2002); *400 ms after*, which was 200–400 ms after sound onset; and *600 ms after*, which was from 400 to 600 ms after sound onset.

Prior to the analyses we checked for outliers in the data. Participants were removed from the datasets when RMSE or RT scores exceeded two standard deviations. The first trial of every condition was treated as a familiarization trial and excluded from the analysis. Pairwise comparisons were made using Bonferroni correction (*α* = 0.001), and Greenhouse–Geisser correction was used when sphericity was violated.

We use subscripts to denote the specific conditions of the STs and DTs. For example, we use DT_200_ to denote a DT with 200-ms predictability or ST_400_ to denote an ST with 400-ms predictability. DT costs (DT_cost_) were calculated with the formula [(RMSE_ST_ − RMSE_DT_)/RMSE_ST_] × 100 (Bock 2008).

RMSE and RTs were submitted to 2 × 5 repeated-measures analyses of variance (ANOVAs) with the factors Task Type (ST vs. DT) and Predictability (0 ms vs. 200 ms vs. 400 ms vs. 600 ms vs. 800 ms). Velocities were analyzed with a 5 × 4 repeated-measures ANOVA with the factors predictability (0 ms vs. 200 ms vs. 400 ms vs. 600 ms vs. 800 ms) and interval (200 ms before vs. 200 ms after vs. 400 ms after vs. 600 ms after sound onset).

### Results

#### Questionnaire

Of the 35 participants, two thirds stated that they did not pursue any specific DT strategy; the other third prioritized tracking over tone response. When asked about their preferred predictability condition, 28.6% chose 800 ms, 40.0% chose 600 ms, 25.7% chose 400 ms, and 2.85% each chose 200 ms and 0 ms. Some participants verbally reported that they felt distracted by too much visual information (cf., de Oliveira et al. 2014). Participants reported that the 600 ms predictability was most helpful although their best performance was at 400 ms.

#### Visuomotor tracking task

##### RMSE

**Fig. 3:**
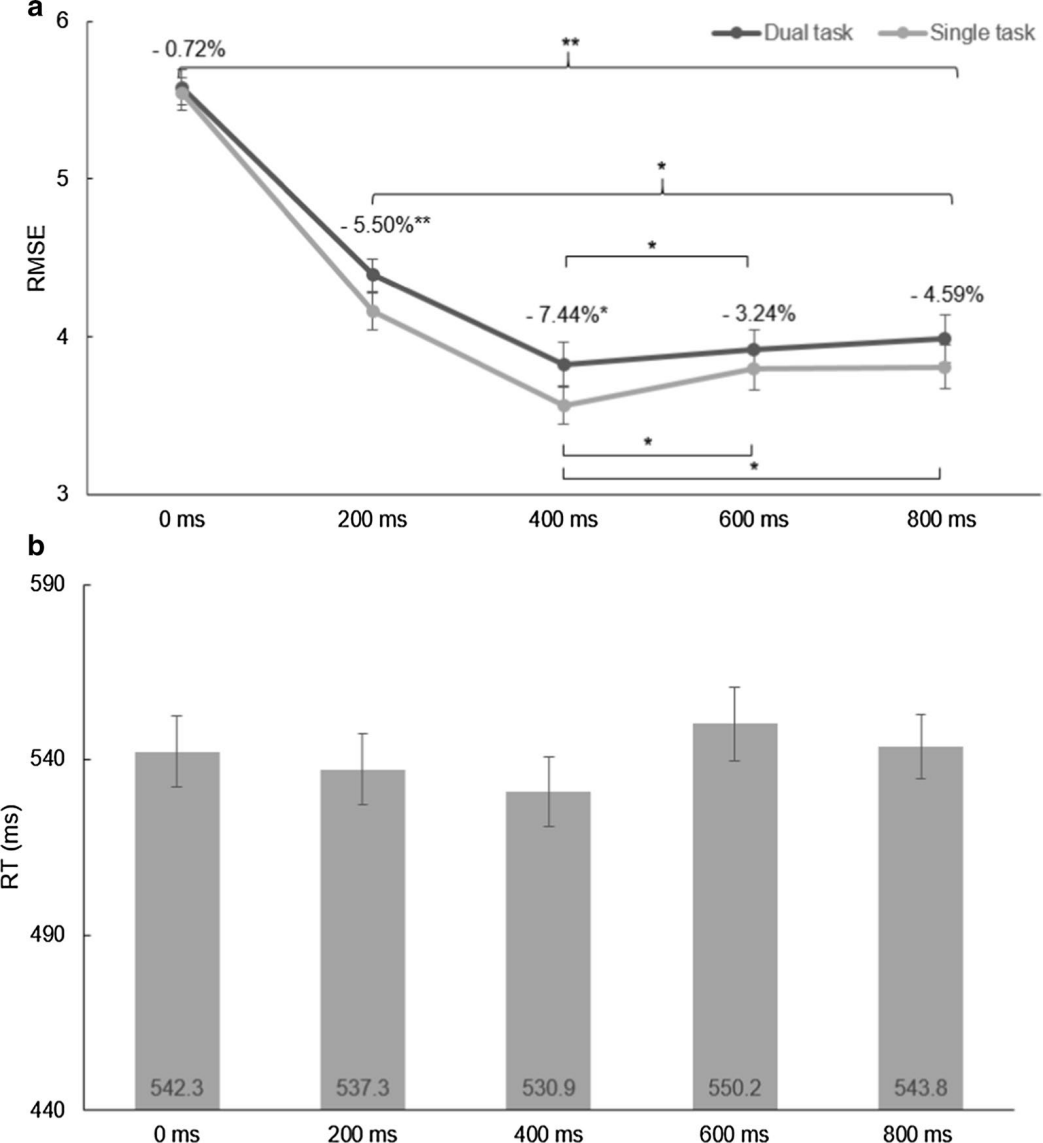
Performance on the 6 predictability conditions. **a** Tracking performance in Experiment 1 as indicated by root-mean-square error (RMSE). The light gray line represents mean RMSE for dual-task conditions, the dark gray line single-task conditions. Asterisks denote significant differences between single- and dual-task conditions. Dual-task costs, which are added to the graph as percentages, were significantly reduced in the 200-ms and 400-ms conditions. **b** Reaction times in dual-task conditions. Asterisks denote significant differences between predictability conditions. Conditions varied in predictability (i.e., length of the visible path) from 0 to 800 ms. In both panels, error bars show the standard error

There was a significant main effect of task type, *F*(1, 34) = 11.63, *p* = 0.002, *η*^2^ = 0.255, because participants were better in single-task tracking, and there was a significant effect of predictability, *F*(4, 136) = 165.62, *p* < 0.001, *η*^2^ = 0.830, with RMSE being lowest in the 400-ms predictability condition. There was no significant interaction, *F*(4, 136) = 0.69, *p* = 0.597, *η*^2^ = 0.020. There were significant differences between 0 ms and all conditions containing visual information, as well as between 200 ms and the remaining visual conditions, in both single- and dual-task trials. There were no significant differences between 600 and 800 ms (Fig. 3a). Looking further into those conditions that contained visual information (200–800 ms), we found that the relationship between predictability and RMSE was best described by a quadratic function, *F*(1, 34) = 26.80, *p* < 0.001.

##### Velocities

**Fig. 4:**
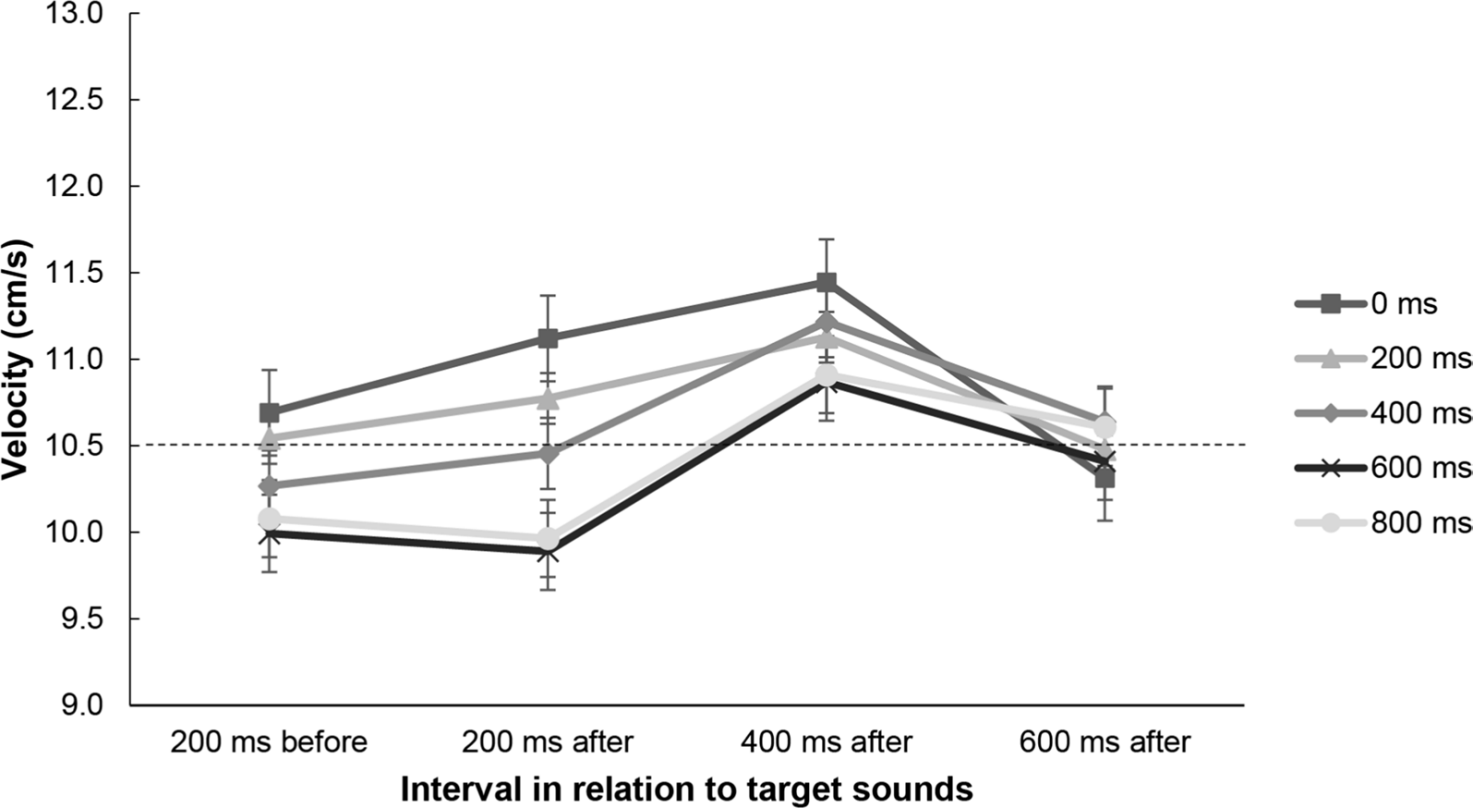
Results of velocity analyses in Experiment 1. The dashed horizontal line represents the constant target velocity (10.5 cm/s). Baseline tracking velocity (i.e., 200 ms before the sound onset) was compared against 200 ms, 400 ms, and 600 ms after the sound onset. Error bars show the standard error. Different symbols represent the different predictability conditions

The repeated-measures ANOVA revealed a significant main effect of predictability, *F*(4, 124) = 7.81, *p* = 0.036, *η*^2^ = 0.079, because there was a tendency toward faster tracking with less visual information (0 and 200 ms) and slower tracking with more visual information (600 ms and 800 ms). There was a significant main effect of interval, *F*(23, 93) = 16.71, *p* < 0.001, *η*^2^ = 0.350, because in all visual predictability conditions participants were fastest in the interval of 400 ms after sound onset (Fig. 4). There was also a significant Predictability × Interval interaction, *F*(12, 372) = 3.19, *p* < 0.001, *η*^2^ = 0.093, because velocity in the unpredictable condition DT_0_ was furthest from target velocity and velocity in the DT_400_ condition was closest to target velocity.

#### Audiomotor task RTs

There was no significant effect of predictability on RTs, *F*(4, 136) = 2.11, *p* = 0.083, *η*^2^ = 0.058. ST performance for the auditory task was *M* = 464 ms (SD = 52).

##### Errors in the audiomotor task

**Fig. 5:**
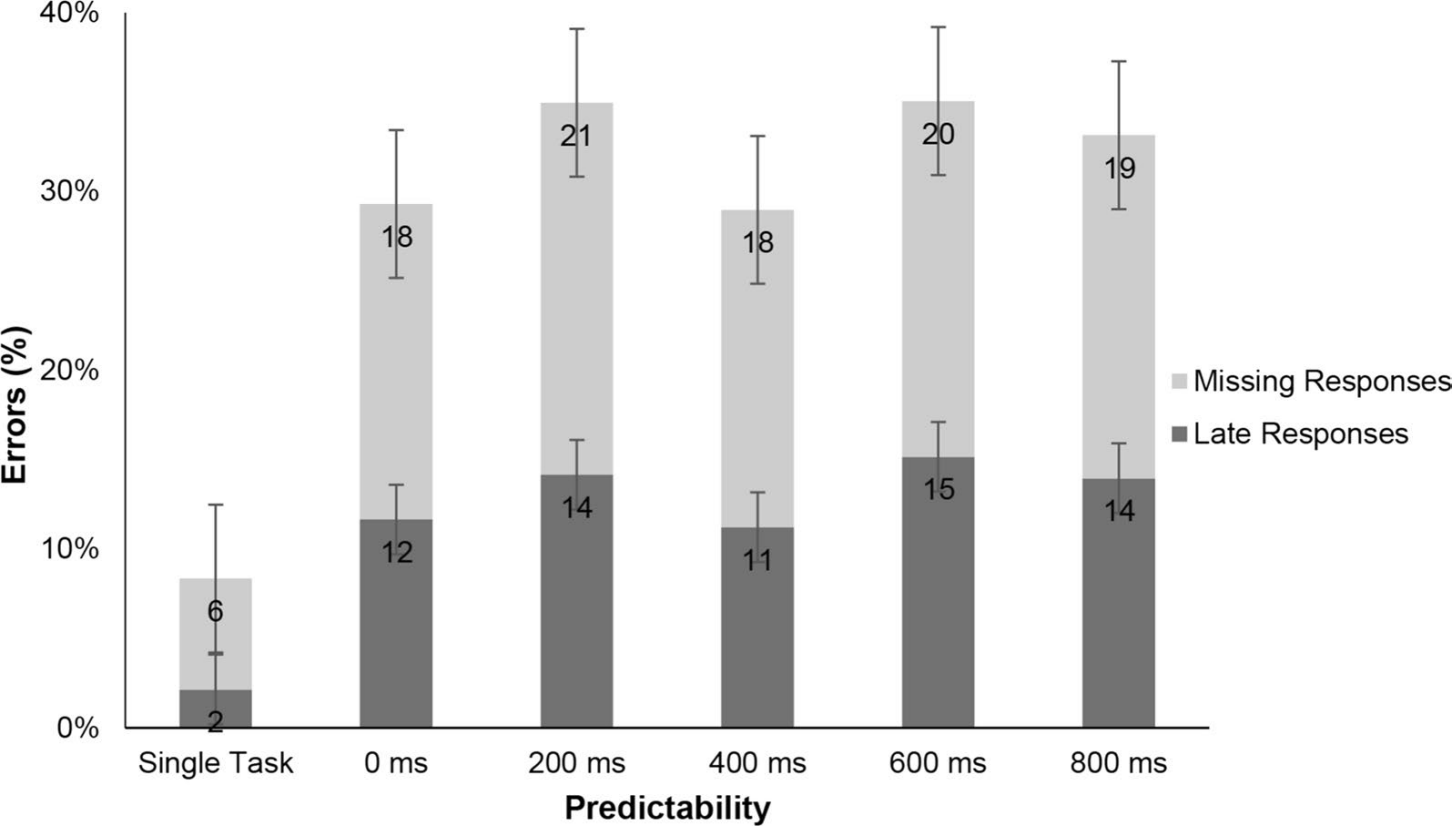
Errors in Experiment 1 were either late responses given later than 800 ms after sound onset (in dark grey) or missing responses that were not given at all (in light grey). Error bars show the standard errors

There were two types of response errors in the auditory task: late responses (Err_late_) were given after the valid period which was between 800 ms after the target sound and the onset of the next sound, or missing responses (Err_miss_) where there was no response between target onset and the following target onset (Fig. 5). There was no significant effect of predictability on late responses, *F*(4, 124) = 1.84, *p* = 0.126, *η*^2^ = 0.056, or on missing responses, *F*(4, 124) = 1.90, *p* = 0.115, *η*^2^ = 0.058. Paired t tests showed significant differences between single- and dual-task error rates (all *t*(31) > 5.56, all *p* < 0.001, all *d* > 0.652).

### Discussion

First, predictability significantly improved visuomotor performance, because dual-task performance improved with visual information. Therefore we conclude that predictability reduces the need for resources. The beneficial effect was most evident for 400 ms, as this was the condition with the lowest RMSE, a more accurate velocity and also lower RT and fewer errors. However, there were no beneficial effects of predictability on secondary task performance, neither on RT nor errors,^2^ which is why we infer that resources were most likely not reinvested. We will discuss the details of these results below.

Regarding the general impact of predictability we conclude that, in line with the basic premise that visual information fosters feedforward control (Weir et al. 1989), predictability enabled more accurate movements in the predictable task. As there was no significant improvement of tracking accuracy beyond 400 ms advance information, this amount of information seems sufficient for performance and optimal for feedforward control as already demonstrated by de Oliveira et al. (2014). It is also in line with research on oculomotor prediction, showing that 500 ms of visual information prior to stimulus occlusion is enough to scale ocular responses (Bennett et al. 2010). It is also in line with research on aiming movements, which demonstrated that people who practiced aiming and were provided with 600-ms vision performed equally well when later provided with only 400 ms (Elliott et al. 1995). This makes (visual) predictability also different from task difficulty. One could have intuitively suggested that with increasing predictability, the task gets simply easier. However, it has been suggested that the relationship between task difficulty and dual-task performance can be described by a linear relationship (e.g., Isreal et al. 1980; McDowd and Craik 1988), but our results suggest that visual information may not be unlimitedly beneficial.

Velocity profiles demonstrated that participants across all conditions showed more speed changes approximately 400 ms after onset of the auditory stimulus. This can be interpreted as DT interference, possibly around response selection, considering that RTs were 540 ms on average. This contrasts with interference found in prior tracking studies, where it typically propagates to *Task 2* and results in longer RTs. Interference in Task 1 can be the result of limbs’ coupling and thus a neuromuscular effect (neural cross-over effect; Wages et al. 2016), an attentional spillover effect (Beilock and Gray 2012), or the result of a strategic timing gain to compensate for the reaction to the sound.

Therefore, regarding our aim to make inferences about resource allocation policy, our results are in line with the notion that visual and auditory tracking tasks draw on the same general pool of resources (Fougnie et al. 2018), because the tracking task seems to have claimed most of the resources, but the peak in velocity demonstrates that a share of resources was temporarily allocated to the audio task to prepare pedal responses. This share of resources satisfied the minimum requirement of giving a response, yet it seems that not enough residual resources were invested to actually reduce reaction times and improve secondary task performance. According to modality-specific resource accounts (Wickens 2008), resources utilized for a visual task should not interfere with demands from an auditory task and thus could not explain the increased tracking velocity. The velocity change was most pronounced in the 0-ms condition, which was the condition without any predictive component and therefore fundamentally different from the other conditions. It seems that the constantly changing environment forced participants to overtake and drop back behind the target more often, which resulted in more overall velocity changes, reflecting the highest need for resources (as also mirrored by no differences between ST and DT performance in RMSE). In contrast, the effect was least pronounced in the 600- and 800-ms conditions, suggesting forward control in response to the upcoming path (Hill and Raab 2005) which enabled participants to stay closely behind the target, without the need for constant alignment around the target, and possibly less need for resources.

Another possible explanation for the results is that the increased share of resources to the visually predictable task might be the result of task prioritization. It is plausible that more resources were allocated to the task that was most achievable, which would be in line with increasing error rates for conditions where visual information was present.

## Experiment 2a

Experiment 1 showed that participants’ performance improved in the predictable task but not in the secondary, unpredictable task. It seems that most of the resources, drawn from one general pool of resources, were allocated to the predictable task but that residual resources freed by predictability were not reinvested into the secondary task. In Experiment 2, we turned the manipulations around by making the secondary task predictable and leaving the continuous task unpredictable, and examined resource allocation policies for a predictable secondary task.

*Prior knowledge*, as the second source of predictability (Wolpert and Kawato 1998), can be induced via sequences in discrete tasks. Sequences and regularities increase the likelihood of stimulus occurrence and reduce uncertainty about stimulus onset, which enables participants to respond in a timely fashion (Capizzi et al. 2012; Nobre et al. 2007; Requin et al. 1991; Rolke and Hofmann 2007). In line with the argument presented above, this should result in enhanced accuracy, considerably reduced RTs, and fewer attentional resources required (de la Rosa et al. 2012). Töllner et al. (2012) explained that knowledge about a stimulus or task leads to a pre-activation of that sensory modality, freeing up general resources and consequently enhancing encoding and leading to faster response selection. If this holds, sequence learning could lead to faster visual processing and shorter motor response execution times in visuomotor tasks (De Jong 1995; Sigman and Dehaene 2006). In fact, two DT studies (Cutanda et al. 2015; de la Rosa et al. 2012) demonstrated that regular auditory sequences led to faster reaction times, equally effective in ST and DT conditions and irrespective of high or low load in the working memory task of the DT condition. However, RTs for ST and DT performance of the secondary working memory task were not explicitly contrasted and allocation policies could not be inferred.

### Methods

#### Participants

For Experiment 2a, we recruited 24 participants. Two participants were excluded from the analyses because testing was terminated due to a technical malfunction, yielding a final sample of 22 participants (10 males and 12 females; aged between 18 and 30 years, *M* = 22.82 years, SD = 3.20). As Experiment 1 showed stable performance on the tracking task after very few trials, and thus high correlations among trials [DT_0_: Cronbach’s *α* = 0.927 (mean correlation among trials: r = 0.674), DT_200_: *α* = 0.935 (r = 0.648), DT_400_: *α* = 0.959 (r = 0.769), or DT_600_: *α* = 0.943 (r = 0.669)], the a priori sample-size estimations for Experiment 2 were adapted: *α* = 0.05, 1 − *β* = 0.80, *r* = 0.7 (G*Power 3.1.9.2). This revealed a test power of 0.81 and a required sample size of 22 participants.

#### Setup

The setup was the same as in Experiment 1. We used a 16-bit joystick (Thrustmaster T16000M FCS, max sampling rate 120 Hz).

#### Manipulation of predictability

##### Visuomotor tracking task

The task and display were the same as in Experiment 1, but only the unpredictable 0-ms condition was applied.

##### Audiomotor task

**Fig. 6:**
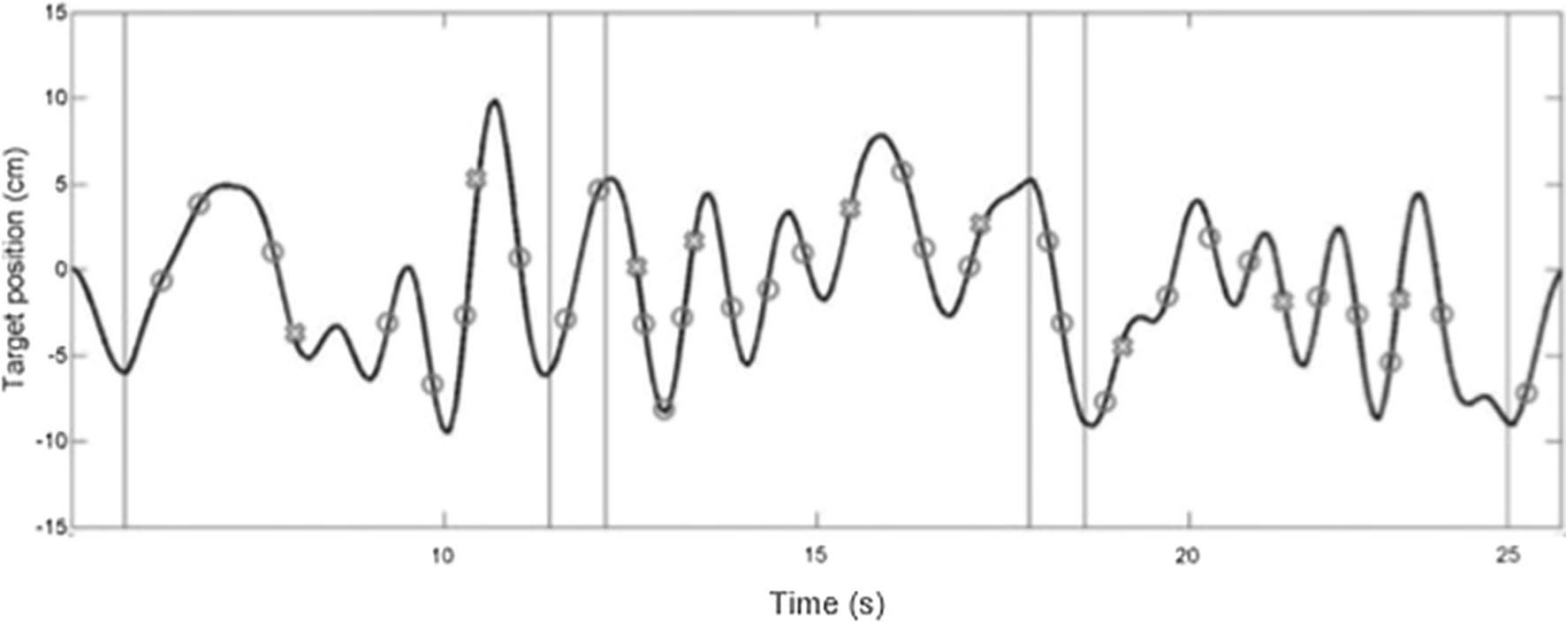
An example of a sequenced dual-task trial in Experiment 2a. A tracking target followed the sinusoidal path, which was invisible to the participants. Circles along the tracking path represent the occurrence of distractor sounds; crosses along the tracking path represent target sounds. All sounds had varying inter-stimulus intervals. The only regularity in the predictable condition was the occurrence of a target sound every fourth sounds

The secondary task was an auditory discrimination task. In the predictable/sequenced condition, tones were arranged in a sequence with every fourth sound being the high-pitched target sound (see Fig. 6) with varying inter-stimulus intervals ranging between 750 and 1,050 ms. In the unpredictable/random condition, high- and low-pitched tones occurred randomly with the same varying inter-stimulus intervals. The number of target sounds per trial varied between 9 and 12 in unpredictable conditions where sounds occurred randomly (every 1.9 to 3.0 s, following Raab et al. 2013).

#### Procedure

After the familiarization phase, participants took about 30 min to perform 50 trials. Participants began with 10 ST tracking trials, after which they completed four more blocks that were randomized across participants: 10 ST auditory trials with random sounds, 10 ST auditory trials with sequenced sounds, 10 DT trials with random sounds, and 10 DT trials with sequenced sounds.

#### Data analysis

As in Experiment 1, we calculated the average RMSE and tracking velocities as a measure of tracking performance and RTs plus errors as a measure of performance in the audiomotor task. We used rand to denote trials with randomly occurring sounds and seq to denote trials with sequenced sounds (e.g., DT_rand_, ST_seq_).

The RMSE were compared between ST and DT trials with two paired-*t* tests (ST vs. DT_rand_; ST vs. DT_seq_). Further, for DT trials, RMSE was submitted to a one-way repeated-measures ANOVA with factor Sound Order (random vs. sequenced). Velocities were analyzed with a 2 × 4 repeated-measures ANOVA with factors Sound Order (random vs. sequenced) and Interval (200 ms before sound onset vs. 200 ms after onset vs. 400 ms after onset vs. 600 ms after onset). RTs were submitted to a 2 × 2 repeated-measures ANOVA with the factors Sound Order (random vs. sequenced) and Task Type (ST vs. DT).

### Results

#### Visuomotor tracking task

##### RMSE

There was no effect of sound order on RMSE, *F*(1, 21) = 0.03, *p* = 0.873, *η*^2^ = 0.001. Pairwise comparisons between ST and DT conditions revealed significant differences both when sounds were random, *t*(21) = 3.51, *p* = 0.002, *d* = 0.749, DT_cost_ = − 5.76%, and when sounds were sequenced, *t*(21) = 2.84, *p* = 0.010, *d* = 0.605, DT_cost_ = − 5.44%.

##### Velocities

**Fig. 7:**
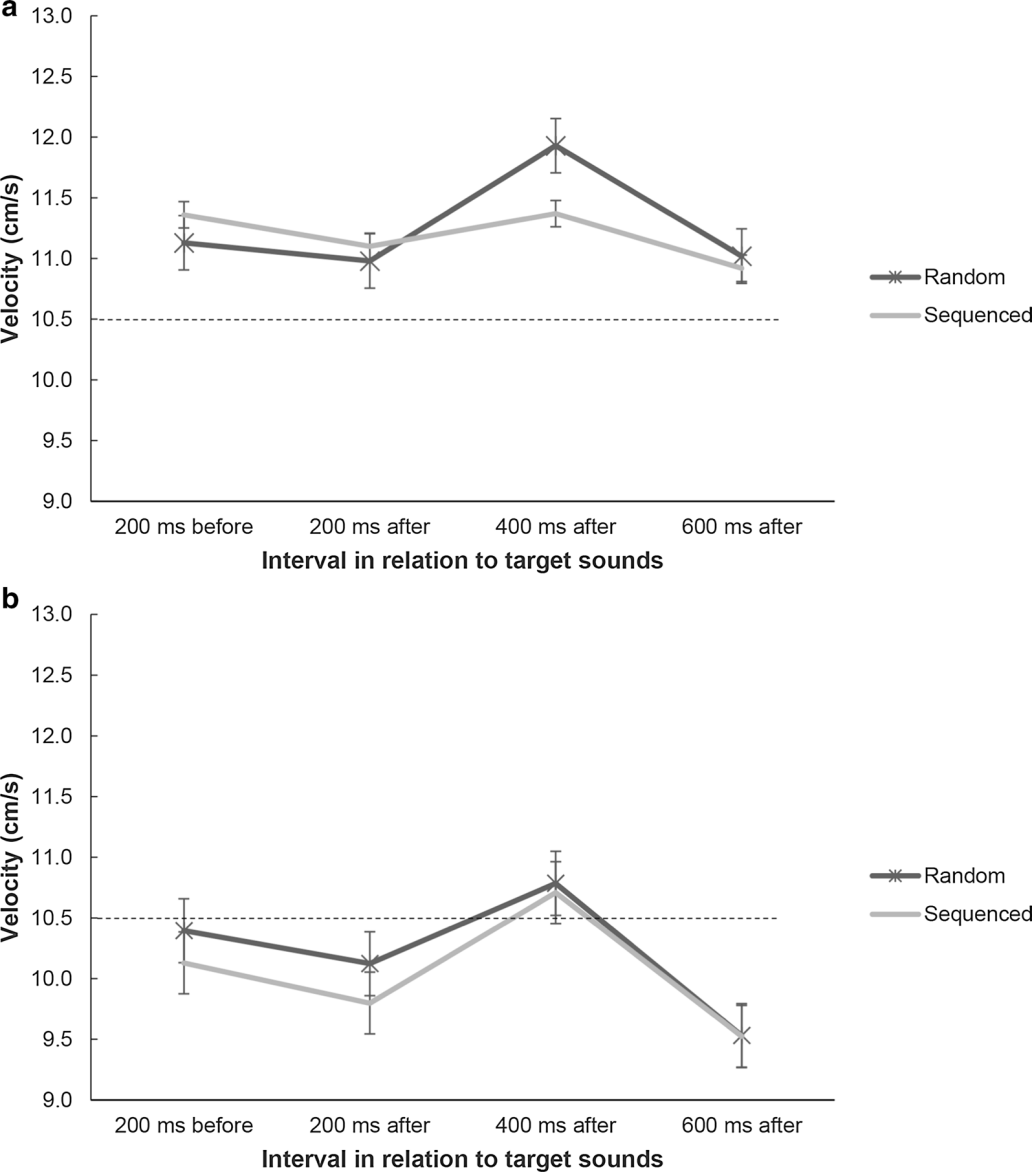
Tracking velocity analyses in Experiments 2a (**a**) and 2b (**b**). Baseline tracking velocity (200 ms before the occurrence of a target sound) was compared against 200 ms, 400 ms, and 600 ms after the sound onset. The dashed horizontal line represents the constant target velocity (10.5 cm/s). Error bars show standard errors

The repeated-measures ANOVA revealed a main effect of interval, *F*(3, 63) = 8.34, *p* < 0.001, *η*^2^ = 0.284, because there was an increase in velocity in the 400-ms after interval. There was also a significant Sound Order × Interval interaction, *F*(3, 63) = 2.87*, p* = 0.043, *η*^2^ = 0.120, because this increase after target sound onset was less pronounced in sequenced compared to random trials (see Fig. 7, top). There was no main effect of sound order on velocity, *F*(1, 21) = 0.44, *p* = 0.516, *η*^2^ = 0.020. In general, participants had higher velocities compared to the target square across all intervals and conditions, which means that the control cursor was ahead of the target square.

#### Audiomotor task RTs

**Fig. 8:**
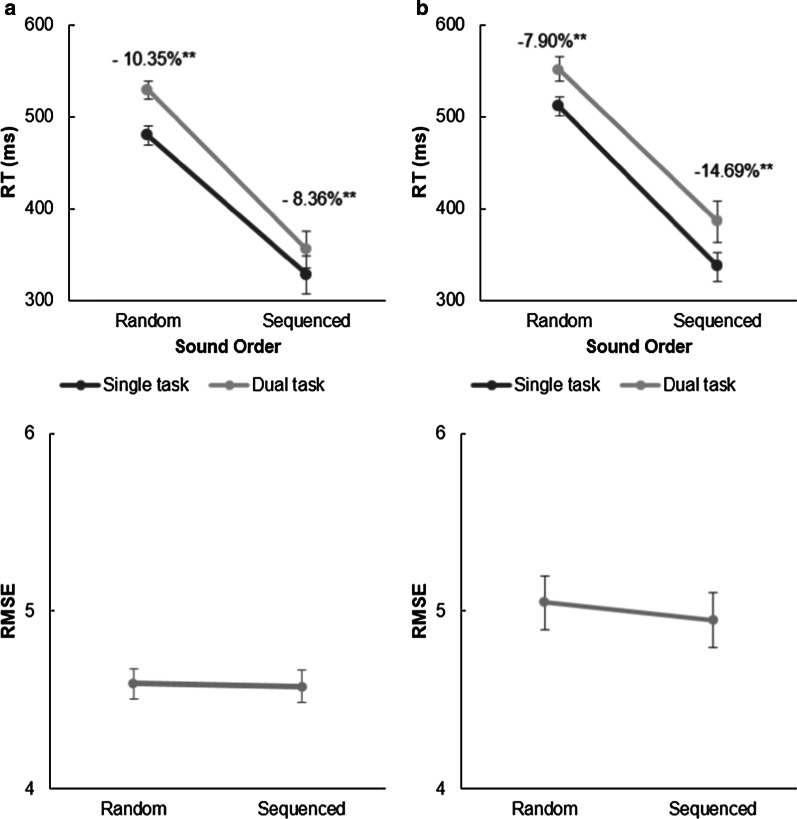
Reaction time (RT) and root-mean-square error (RMSE) analyses in Experiment 2a (**a**) and 2b (**b**). Light gray lines depict dual-task conditions, dark gray lines depict single-task conditions. DT costs are the differences between single- and dual-task conditions, presented as percentages; asterisks denote significant DT costs, **p < .001, *p < .005. Error bars show standard errors

The repeated-measures ANOVA revealed a significant main effect of sound order, *F*(1, 21) = 136.29, *p* < 0.001, *η*^2^ = 0.866, because participants were faster in sequenced compared to random trials. There was also a significant effect of task type, *F*(1, 21) = 28.01, *p* < 0.001, *η*^2^ = 0.571, because participants were faster in ST conditions compared to DT conditions. There was no significant Sound Order × Task Type interaction, *F*(1, 21) = 3.81, *p* = 0.065, *η*^2^ = 0.153 (see Fig. 8). Mean RTs are shown in Table 1.

**Table 1:** Reaction times (in milliseconds) for all conditions in Experiment 2a with the difference between single- and dual-task conditions expressed by dual-task costs (DT_cost_)

Task type	Single task	Dual task	DT_cost_	95% confidence interval	
	*M* (SD)	*M* (SD)		Lower	Upper
Random	479 (50)	529 (47)	− 10.35%**	− 68.020	− 31.019
Sequenced	328 (96)	355 (94)	− 8.36%*	− 47.200	− 7.558

An asterisk denotes significance, **p < .001, *p < .005

##### Errors in the audiomotor task

**Fig. 9:**
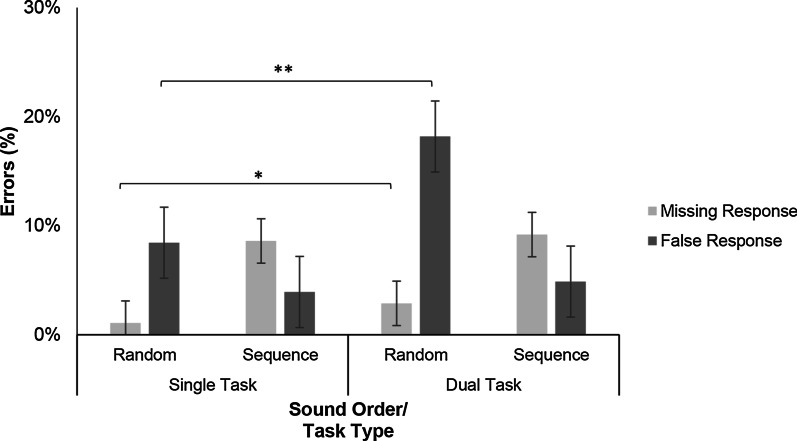
Errors in Experiment 2a were either false responses to distractor sounds or missing responses. There were only significant differences between single- and dual-task conditions when sounds were random, with Err__miss_, *t*(21) = 2.96, *p* = .007, *d* = .711 and Err__false_, *t*(21) = 3.83*, p* < .001, *d* = 967, respectively

There were two types of response errors in the auditory task: false responses when participants pressed the pedal in reaction to distractor sounds, and missing responses (Err_miss_) which did not occur between two consecutive target onsets (Fig. 9). Importantly, responses which were given before sound onset (“premature”) were also counted as missing responses.

There was no significant effect of sound order on false responses, *F*(1, 21) = 2.13, *p* = 0.160, *η*^2^ = 0.092. There was a main effect of task type, *F*(1, 21) = 7.13, *p* = 0.014, *η*^2^ = 0.253, because participants performed better in single-task trials. There was no significant Sound Order × Task Type interaction, *F*(1, 21) = 2.83, *p* = 0.105, *η*^2^ = 0.120.

There was a significant effect of sound order on missing responses, *F*(1, 21) = 6.00, *p* = 0.023, *η*^2^ = 0.222, because participants missed fewer responses in the random conditions. There was no effect of task type on missing responses, *F*(1, 21) = 3.24, *p* = 0.086, *η*^2^ = 0.134, and no significant Sound Order × Task Type interaction, *F*(1, 21) = 3.98, *p* = 0.059, *η*^2^ = 0.159.

Contrary to our expectations, participants failed to react to target sounds and erroneously reacted to distractor sounds more often in sequenced conditions than random conditions (see Fig. 9). As only responses after sound onset were taken into consideration, the high number of missing responses might be explained by premature responses given before sound onset. Therefore, this result is somewhat inconclusive.

When comparing the difference between single- and dual-task conditions, as expected, participants more frequently failed to react to target sounds and falsely reacted to distractor sounds in dual-task conditions compared to single-task conditions.

### Discussion

First, like in Experiment 1, predictability significantly improved dual-task performance, suggesting that predictability reduced the need for resources. Inferring whether performance improvements only emerge in the predictable task and thus a conclusion about whether or not residual resources were reinvested is contingent on the dependent variables as we outline below.

In general, when comparing ST and DT performance in the two conditions, we found typical performance impairment for DT conditions, which was less pronounced for sequenced trials. Sequenced trials lowered RTs to target sounds, lowered DT_cost_, and possibly also reduced the need for resources. This effect occurred even though sequences had varying inter-stimulus intervals making the exact timing of sound onset unpredictable (in contrast to the rhythms used by Capizzi et al. 2012; Cutanda et al. 2015; Halvorson et al. 2013). However, the benefit of sequences was not apparent in the tracking’s RMSE. So while RMSE would not support the hypothesis that residual resources from one general pool were reinvested, velocities show a different pattern. Like in Experiment 1, there were more changes in velocity 400 ms after sound onset—interpretable as interference—but this was not significant for sequenced trials (no significant difference between the intervals 200 ms after and 400 ms after). The velocity analysis would thus suggest that predictability in the auditory task freed enough resources to maintain motor control and accuracy in tracking while preparing pedal responses, defeating, or diminishing interference. Considering that tracking is a continuous task, velocities allow a more fine-grained analysis of performance and interference compared to the exclusively spatial measure RMSE.

## Experiment 2b

Experiment 2a showed that predictability reduced the need for resources, which have been possibly redistributed to tracking in order to maintain motor control during response preparation. Experiment 2b was designed to challenge this finding by increasing cognitive and motor load in the auditory task. To do so, we transformed the go/no-go task into a choice RT task. Participants were no longer required to ignore the low-pitched tones but had to react to both tones with a double pedal, using both feet.

### Methods

#### Participants

For Study 2b, we recruited 24 participants. Four participants dropped out during the experiment, leaving a final sample of 20 participants (15 males and 5 females; aged between 18 and 36 years, *M* = 24.80 years, SD = 4.32).

#### Setup

The setup was the same as in Experiment 2a, except for the foot pedal, which was now a double-foot switch (Scythe USB 2FS-2), fixed centrally under the table.

#### Task and display

##### Visuomotor tracking task

The tracking task and display were identical to those in Experiment 2a.

##### Audiomotor task

The audio task was a choice task, and participants reacted to both tones via the double pedal. They responded to low-pitched (distractor) sounds by pressing the left pedal with the left foot and to high-pitched (target) sounds by pressing the right pedal with the right foot. The sound-order conditions (random and sequenced sounds) remained the same as in Experiment 2a.

#### Procedure

After the familiarization phase, participants took approximately 30 min to complete 50 trials: 10 ST tracking trials, 20 ST auditory trials (10 × random sounds, 10 × sequenced sounds), and 20 DT trials (10 × random sounds, 10 × sequenced sounds).

#### Data analysis

RMSE and velocities were calculated as a measure of visuomotor performance, and RTs were calculated as a measure of audiomotor performance. Differences in RMSE between ST and DT trials were analyzed with two paired-*t* tests (ST vs. DT_rand_; ST vs. DT_seq_). Further, for DT trials, RMSE was submitted to a one-way repeated-measures ANOVA with factor Sound Order (random vs. sequenced). Velocities were analyzed with a 2 × 4 repeated-measures ANOVA with factors Sound Order (random vs. sequenced) and Interval (200 ms before sound onset vs. 200 ms after onset vs. 400 ms after onset vs. 600 ms after onset). RTs were submitted to a 2 × 2 ANOVA with factors Sound Order (random vs. sequenced) and Task Type (ST vs. DT). Errors were subject to a 2 × 2 × 2 ANOVA with factors Sound Order (random vs. sequenced), Task Type (ST vs. DT), and Sound Type (target vs. distractor sound).

### Results

#### Visuomotor tracking task

##### RMSE

There was no effect of sound order on RMSE, *F*(1, 19) = 2.12, *p* = 0.162, *η*^2^ = 0.100. Pairwise comparisons between ST and DT trials revealed deteriorated tracking performance in DTs, both when sounds were random, *t*(19) = 5.91, *p* < 0.001, *d* = 1.322 (ST: *M* = 4.43, SD = 0.35; DT_rand_: *M* = 5.05, SD = 0.68), and when sounds were sequenced, *t*(19) = 4.61, *p* < 0.001, *d* = 0.1031 (ST: *M* = 4.43, SD = 0.35; DT_seq_: *M* = 4.95, SD = 0.68).

##### Velocities

The repeated-measures ANOVA revealed a main effect of interval, *F*(3, 51) = 9.57, *p* < 0.001, *η*^2^ = 0.360 (see Fig. 7, bottom), but there was no main effect of sound order on velocity, *F*(1, 17) = 0.92, *p* = 0.350, *η*^2^ = 0.051, and no significant interaction, *F*(3, 51) = 0.50*, p* = 0.686, *η*^2^ = 0.028.

#### Audiomotor task RTs

The repeated-measures ANOVA revealed a significant main effect of sound order, *F*(1, 19) = 86.33, *p* < 0.001, *η*^2^ = 0.820, because participants were faster in sequenced compared to random trials. There was a significant main effect of task type, *F*(1, 19) = 15.84, *p* < 0.001, *η*^2^ = 0.455, because participants were generally faster in ST conditions than DT conditions, as in Experiment 2a. However, there was no significant Sound Order × Task Type interaction, *F*(1, 19) = 0.16, *p* = 0.690, *η*^2^ = 0.009 (Fig. 7). Mean RTs in the double-pedal experiment are presented in Table 2.

**Table 2:** Reaction times (in milliseconds) for all conditions in the double-pedal experiment (Experiment 2b) with the difference between single- and dual-task conditions expressed by dual-task costs (DT_cost_)

Task type	Single task	Dual task	DT_cost_	95% confidence interval	
	*M* (SD)	*M* (SD)		Lower	Upper
Random	511 (48)	552 (58)	− 7.90%**	− 60.480	− 20.311
Sequenced	336 (69)	386 (102)	− 14.69%*	− 91.600	− 7.135

An asterisk denotes significance, **p < .001, *p < .005

##### Errors in the audiomotor task

There were two types of response errors in the auditory task: false responses when participants used the wrong pedal, i.e., left instead of right pedal for target sounds and right instead of left pedal for distractor sounds; and missing responses (Err_miss_) for target and distractor sounds. As in Experiment 2a, responses which were given before sound onset (“premature”) were also counted as missing responses. There was large percentage of false responses to target sounds, most likely due to a large amount of premature responses (as they were counted in the interval after distractor sounds). We therefore decided not to consider errors further, but details can be seen in appendix.

### Discussion

In Experiment 2b we showed that predictability had a positive impact on audiomotor performance, even though this effect was less pronounced than in Experiment 2a. Whereas in Experiment 2a the impact of predictability on tracking performance seemed to be dependent on the variable examined, results of Experiment 2b were more clear-cut. There was neither a positive impact on RMSE nor a less pronounced velocity increase for sequenced conditions. We conclude that auditory predictability was strong enough to buffer load induced by simple reactions (Experiment 2a), but that more complex choice reactions require additional resources that could not be reinvested in tracking (possibly because they include the excitation of different hemispheres and the initiation of motor action in different limbs). Note that Experiment 2b included fewer participants than planned and this may put into question the nonsignificant results obtained; this is a limitation of this study. However, given the effect sizes and significant results obtained in the experiment we believe the sample size was adequate for the statistical analysis done.

In sum, Experiments 1 and 2 showed that predictability reduced the need for resources; visual predictability reduced the need for resources in tracking and auditory predictability reduced the need for resources in audiomotor reactions. As there were no improvements in the unpredictable task, it seems unlikely that residuals were reinvested; however, velocity profiles speak for one general rather than modality-specific pools of resources.

It is possible that participants did not reinvest residuals because the two tasks were unrelated. Naturally, participants invested more resources in the tracking task because of its continuous nature. The auditory task was therefore always disruptive, irrespective of whether it was predictable, and required fewer resources. Hence, there may have been little incentive to invest in a disruptive task. If, however, the distractive task was transformed into a helping task, then this could be an incentive for reinvestment. This could be achieved by having one task predict changes in the other task. Therefore, in Experiment 3 we examined the role of task structure and between-task predictability in resource allocation policies.

## Experiment 3

So far, Experiments 1 and 2 showed that predictability positively influences dual-task performance, predominantly through improvements in the predictable task. While this result per se could have questioned a general pool of resources, velocity analyses have shown that the auditory task takes away some of the resources from tracking and thus support the generic resource assumption. Yet our data did not support reinvestment of resources into a secondary task.

Wahn and König (2015, 2017) argued that resource allocation can be task-dependent and that while object-based vs. spatial tasks (visual and auditory) partially share resources, two spatial tasks (visual and auditory) fully share resources. If this is true, then adding a spatial component to the auditory discrimination task in our study, should enable resource reinvestment. We therefore placed target sounds 250 ms before inflection points of the curve and hypothesized that this would decrease the need for resources and enable participants to reinvest resources. Similar approaches have been taken by task integration studies that covaried two tasks (e.g., de Oliveira et al. 2017). Schmidtke and Heuer (1997) showed for instance that sequences could be more easily implemented when they were temporally correlated with another discrete task. Likewise, de Oliveira et al. (2017) also positioned target tones 250 ms before inflection points of a tracking path, so that participants could relate the occurrence of a tone to a motor action and found that participants in the covariation group showed significantly better performance in DT than in ST. This effect was pronounced not only in repeating segments of the curve but also in random outer segments, suggesting that covariation can facilitate performance even in otherwise unpredictable environments.

### Method

#### Participants

We recruited 22 participants. After we removed one person as an outlier, the final sample consisted of 21 participants (11 males and 10 females; aged between 19 and 35 years, *M* = 23.90 years, SD = 3.49). Sample size estimations were based on Experiment 2 (i.e., *α* = 0.05, 1 − *β* = 0.80, *r* = 0.7, test power of 0.81 and a required sample size of 22 participants).

#### Setup

The setup of Experiment 3 was the same as in Experiment 1.

#### Task and display

##### Visuomotor tracking task

The tracking task and display were identical to those in the other experiments, but the tracking path was calculated using a different formula. To guarantee enough distance between sounds and curves, the new paths were stretched out. They were composed of three segments, each obeying the formula:

$$f\left( x \right) = b_{0} + a_{1} \sin \left( {i \times x} \right) + b_{1} \cos \left( {i \times x} \right) + a_{2} \sin \left( {i \times x} \right) + b_{2} \cos \left( {i \times x} \right) + a_{3} \sin \left( {i \times x} \right) + b_{3} \cos \left( {i \times x} \right)$$

with *a*_*i*_* and b*_*i*_ being randomly generated numbers ranging from − 10 to 10 and *x* being a real number in the range [0; 2π].

##### Audiomotor task

Participants responded to high-pitched sounds by pressing on a pedal. High-pitched sounds always occurred 250 ms before a turning point in the tracking curve (integrated conditions); low-pitched sounds occurred randomly between these events and did not require a response by the participant.

#### Procedure

After the familiarization phase (DT familiarization with random sounds), participants took about 35 min to perform 60 trials: 20 ST tracking trials, 20 ST auditory trials, and 20 DT trials (10 × random, 10 × integrated). After completing the experiment, participants answered a questionnaire that contained five questions designed to gradually reveal participants’ knowledge of the manipulation. The primary purpose of this questionnaire was to label participants with “knowledge” vs. “no knowledge,” so that knowledge could be entered as a between-subjects factor (see Data Analysis). We first asked whether they had noticed anything special during the experiment, then whether they felt supported or distracted in some of the DT conditions, and then whether they had detected any regularities. After this, participants were told that high-pitched tones served to indicate changes in tracking and were asked whether they had noticed this. If participants answered yes, the fifth question asked them how the tone indicated changes.

#### Data analysis

For Experiment 3 we use DT_cov_ for DT trials where tracking and auditory task covaried (i.e., stimuli could be integrated) and DT_rand_ for random sounds. We compared the RMSE between ST and DT trials with two paired *t* tests (ST vs. DT_rand_; ST vs. DT_cov_). Further, for DT trials, RMSE was submitted to a one-way repeated-measures ANOVA with the factor Sound Location (random vs. covariation). Velocities were analyzed with a two-way repeated-measures ANOVA with the factors Sound Location (random vs. covariation) and Interval (200 ms before onset vs. 200 ms after onset vs. 400 ms after onset vs. 600 ms after onset). RTs were submitted to a 2 × 2 ANOVA with the factors Sound Location (random vs. covariation) and Task Type (ST vs. DT). Knowledge about the task integration was entered into the analysis as a between-subjects factor.

### Results

#### Questionnaire

Participants were classified as having knowledge about the manipulation when they were able to correctly describe the task integration manipulation in the fifth question. In total, 10 participants (47.62%) were able to verbalize the positioning of sounds in the questionnaire after finishing the experiment.

#### Visuomotor tracking task.

##### RMSE

**Fig. 10:**
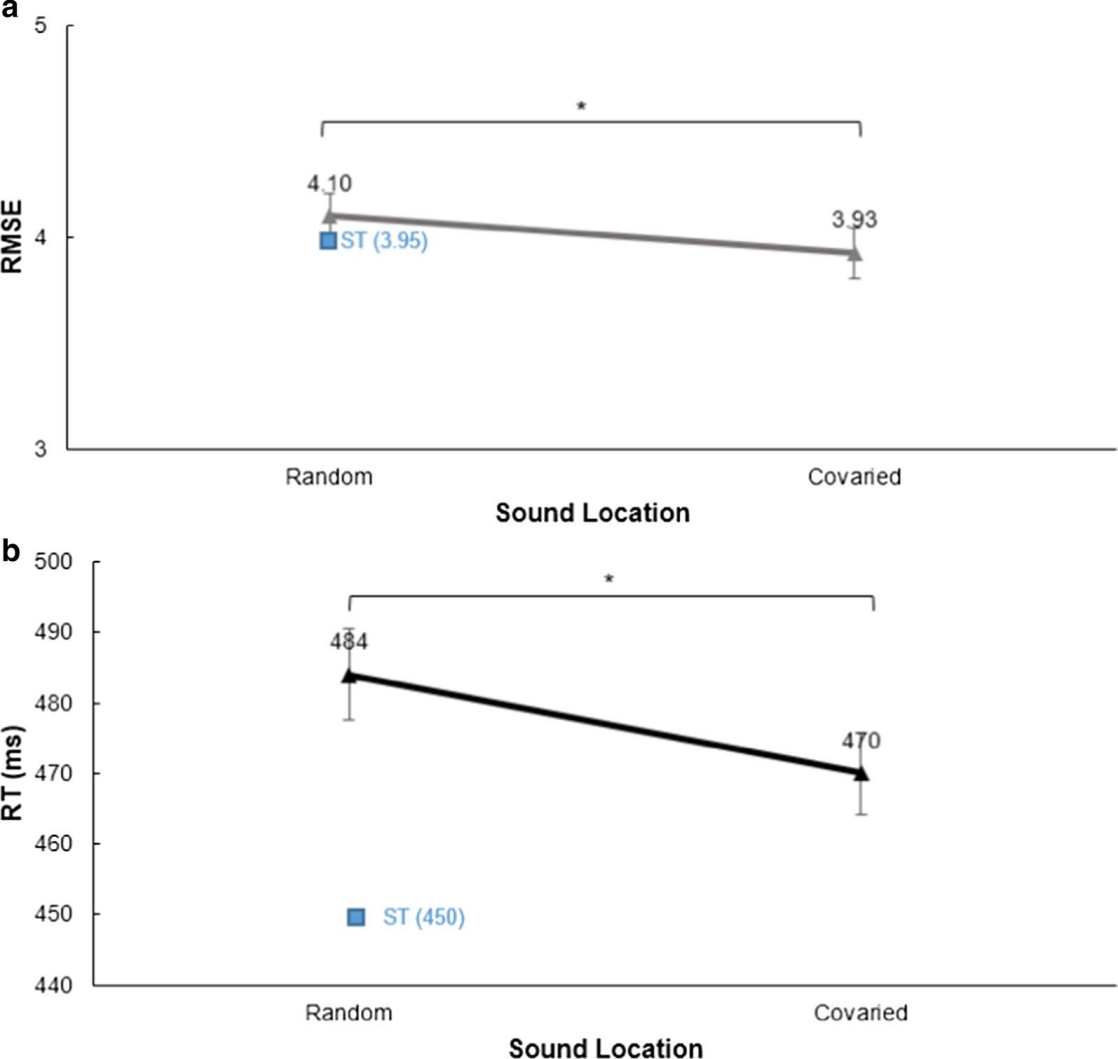
Performance in Experiment 3 by covaried or random sound location. **a** Results of tracking performance for dual-task conditions in Experiment 3 as indicated by root-mean-square error (RMSE). **b** Reaction times (RTs) in milliseconds in dual-task conditions. In both panels, single-task (ST) performance is depicted by a single data point represented by a square

There was a significant main effect of sound location on RMSE, *F*(1, 20) = 5.46, *p* = 0.030, *η*^2^ = 0.214, because participants showed better tracking performance when sounds were indicative of turns in the tracking task (DT_cov_: *M* = 3.93, SD = 0.54; DT_rand_: *M* = 4.10, SD = 0.47; ST_rand_: *M* = 3.95, SD = 0.49; Fig. 10). Participants who acquired knowledge about the manipulation did not show better tracking performance, Sound Location × Knowledge, *F*(1, 19) = 2.28, *p* = 0.148, *η*^2^ = 0.085.

##### Velocities

**Fig. 11:**
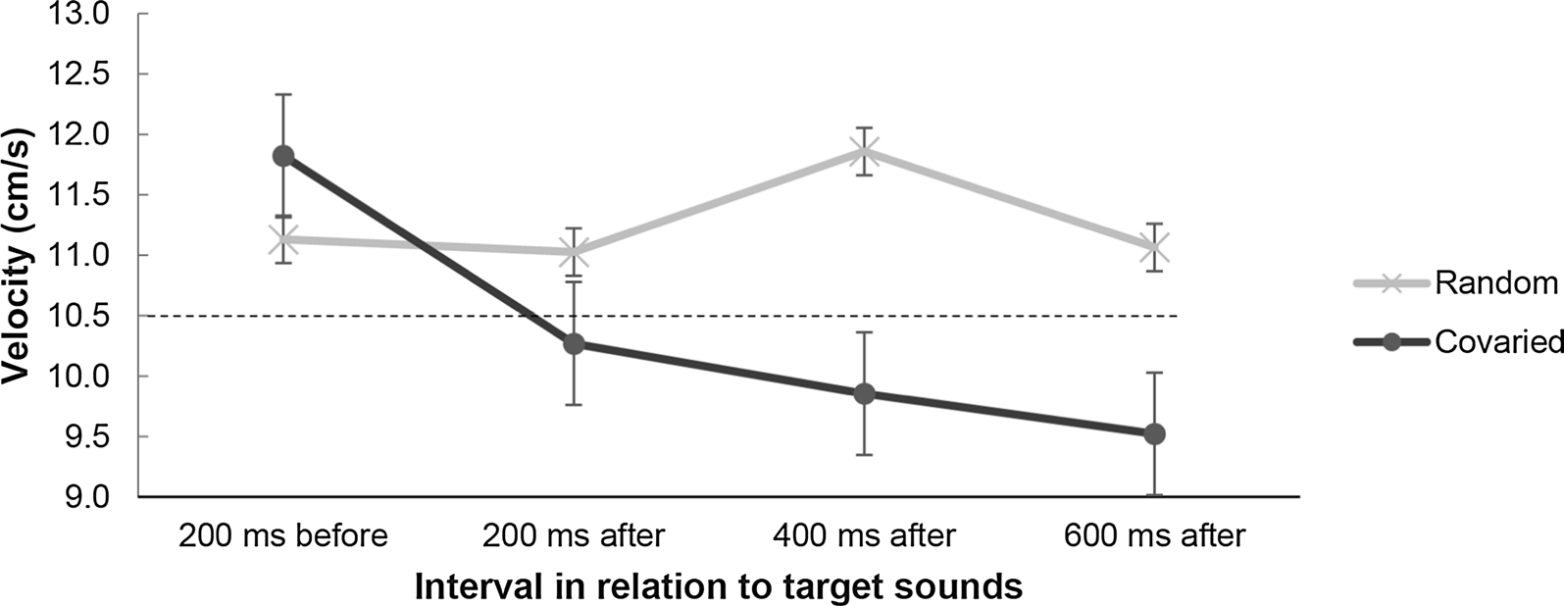
Velocity analyses in Experiment 3. Baseline tracking velocity (200 ms before the occurrence of a target sound) was compared against 200 ms, 400 ms, and 600 ms after the sound onset. The dashed horizontal line represents the constant target velocity (10.5 cm/s). Covaried refers to dual-task trials in which sounds were coupled to the tracking path. Error bars show standard errors

For tracking velocities, there were main effects of sound location, *F*(1, 20) = 46.14, *p* < 0.001, *η*^2^ = 0.698, and interval*, F*(3, 60) = 23.74, *p* < 0.001, *η*^2^ = 0.543, as well as a significant interaction, *F*(3, 60) = 21.83, *p* < 0.001, *η*^2^ = 0.522. For random conditions the velocity pattern was similar to that in Experiments 1 and 2, but for integrated conditions there was a very different pattern as participants slowed down after target sound onset (see Fig. 11).

#### Audiomotor task RTs

There was a main effect of sound location on RTs, *F*(1, 20) = 6.59, *p* = 0.018, *η*^2^ = 0.248 (Fig. 10), showing that participants reacted significantly faster when sounds covaried with the tracking path (DT_rand_: *M* = 483 ms, SD = 30; DT_cov_: *M* = 470 ms, SD = 26; ST_rand_: *M* = 449 ms, SD = 34). Knowledge about the location of sounds did not affect RTs, Sound Location × Knowledge, *F*(1, 19) = 0.32, *p* = 0.581, *η*^2^ = 0.012.

##### Errors in the audiomotor task

**Fig. 12:**
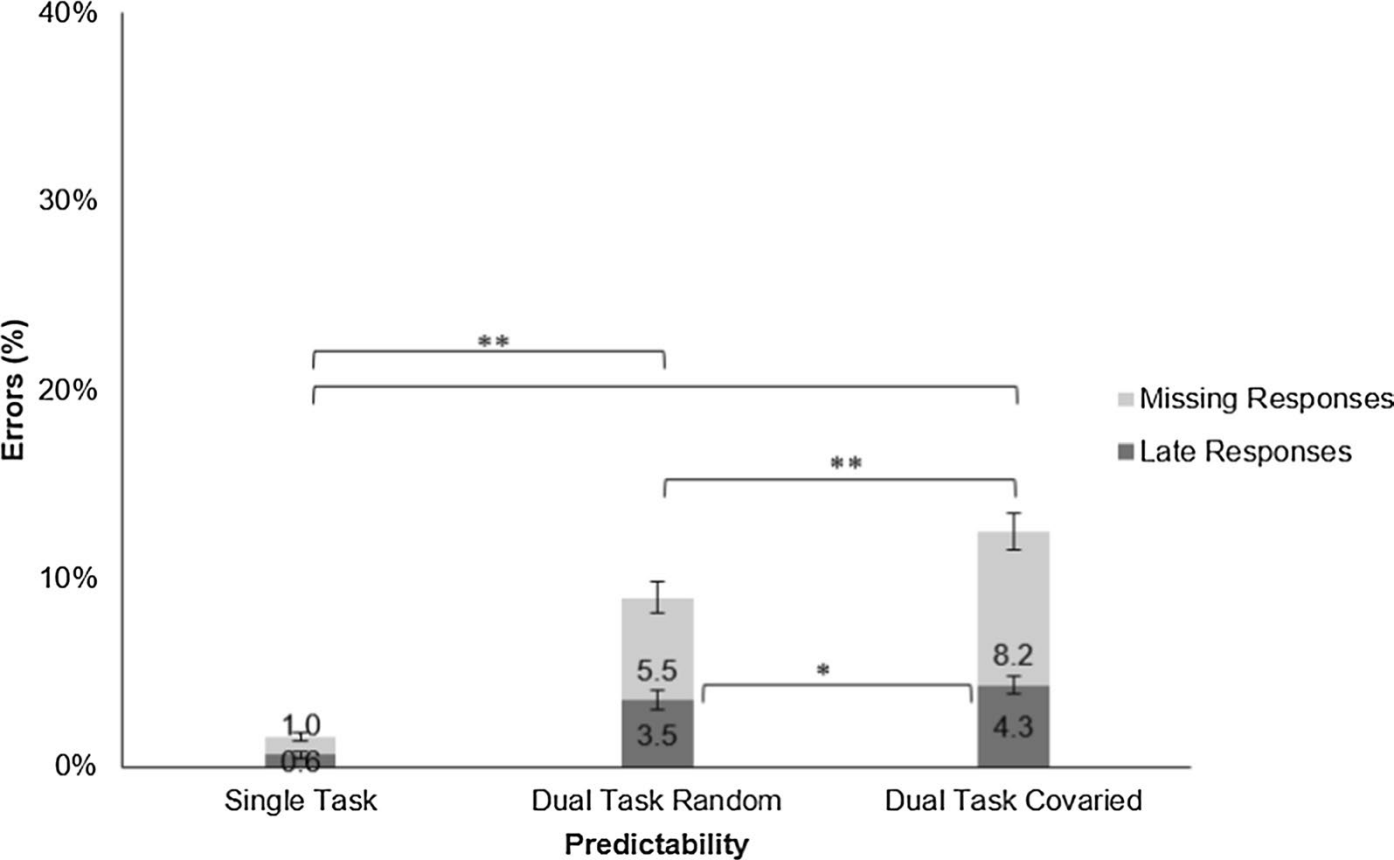
Errors in Experiment 3 were either late responses given later than 800 ms after sound onset (in dark grey) or missing responses that were not given at all (in light grey). Error bars show standard errors

There were two types of response errors in the auditory task: late responses (Err_late_) which were given after the valid period (from 800 ms after the target sound until onset of the next sound), and missing responses (Err_miss_) where there was no response between two consecutive target onsets (Fig. 12). There was a significant main effect of predictability on late responses, *F*(1, 20) = 5.19, *p* = 0.034, *η*^2^ = 0.034, and on missing responses, *F*(1, 20) = 26.96, *p* < 0.001, *η*^2^ = 0.100, showing that errors were larger when the tasks covaried. Paired *t* tests showed significant differences between single- and dual-task error rates (all *t*(20) > 7.21, all *p* < 0.001, all *d* > 1.47), as well as between the random and covaried dual-task conditions (late responses: *t*(20) = 2.28, *p* = 0.034, *d* = 0.497; missing responses: *t*(20) = 5.19, *p* < 0.001, *d* = 1.132).

### Discussion

In Experiment 3 we found a beneficial effect of predictability on performance in both tasks, which is in contrast with Experiments 1 and 2. Participants had faster reaction times and also fewer tracking errors. Further, they decreased velocity after sound onset, showing that they probably learned to drop back behind the target and prepare motor responses as soon as the sound had announced upcoming changes in the tracking curve. It is conceivable that the target sounds and their clear spatial location thus served as a warning signal, which in turn eased resource allocation. As the sound was no longer intrusive but helpful, resources could be more easily allocated and shared between tasks, which is why we conclude that covariation between tasks improves DT performance by fostering resource reinvestment. Future research could examine possible mechanisms underlying this effect. As suggested by Künzell et al. (2018) and Koch et al. (2018), covariation and a shared higher-level goal can prompt participants to treat two tasks as one integrated task (see also Schmidtke and Heuer 1997). One such (implicit) goal or action might have been “turn after pressing” rather than the separate goals of “pedal press” and “track the cursor,” but further measures are needed to test such conceptualization mechanisms. Whether the conceptualization is implicit or explicit does not seem to matter, given that the one half of our subjects which was able to verbalize the position of the sound, performed as well as those without explicit knowledge.

## General discussion

The purpose of our experiments was to examine the impact of predictability on dual-task performance and gain insight into resource allocation policies. Experiments 1 and 2, which manipulated predictability in either the first or the second task, showed that performance improved in the predictable task, but that residual resources were not reinvested in the other task. This is in line with economical processing accounts (Navon and Gopher 1979). In contrast, Experiment 3 covaried two tasks by including an auditory element in the tracking task (and conversely, a spatial element in the auditory task). The results show clear performance improvements in both tasks and thus possibly better resource sharing and reinvestment across tasks. We therefore conclude that predictability helps to circumvent attentional resource limitations (cf., Wahn and König 2017, p. 91) and that the extent to which resources can be shared among tasks depends on the tasks and their characteristics [see also the claim by Tombu and Jolicœur (2003, p. 4), that “determining exactly which task characteristics affect capacity allocation is an empirical issue that will need to be resolved”].

Overall, our results contribute to the ongoing debate about whether limited resources are specific to modalities. Our findings lend support to the theory of general resources rather than modality-specific resources. It is possible that predictability freed up modality-specific resources that could not be reinvested into the other-modality task. However both the velocity profiles in all Experiments and the results of Experiment 3 (an auditory cue aiding visuomotor performance) demonstrate that the visual tracking task and the auditory RT seem to draw on common central attentional resources. Because velocity data demonstrate that participants are able to continue tracking while responding to sounds [i.e., called hesitations in Klapp et al. (1987) and Tsang and Chan (2015)], this strengthens the basic premise that parallel processing and execution is possible. However, dual-task costs showed a small impact of the secondary task, so it is possible that the tracking task demands constant resources and a certain share is always taken by this task. If we consider the concurrent use of hand and foot as same-modality response, the results further strengthen the hypothesis that interference occurs when tasks draw on the same resources (Meyer and Kieras 1997; Wickens 2002). Consistent velocity increases around pedal responses suggest interference at response-activation or execution stages, because motor-related resources would have to be taken away from manual tracking. It has been suggested that such cross-talk can be overcome with practice by integrating two tasks (Bratzke et al. 2009; Heuer and Schmidtke 1996; Swinnen and Wenderoth 2004), which would also be substantiated by the findings of Experiment 3.

An alternative explanation for our findings concerns task prioritization. Wickens et al. (2015) suggested in their strategic task overload management model that some task characteristics such as salience can foster the prioritization of a task. It is possible that participants did not reinvest resources into the other task in Experiments 1 and 2 because predictability prompted a shift in priority toward the predictable task. In a dual-task learning experiment (Broeker et al. 2020a), participants performed the tracking task with a constant middle segment (random outer segments) for two days. One group was informed about the repeating segment, the other group was supposed to acquire implicit motor knowledge. On day three, visual information (400 ms) was added to the tracking task. Results showed an additive effect of knowledge and visual information, meaning that both sources of predictability independently improved tracking performance, but importantly, reaction times did not improve. Capacity-sharing accounts support the notion that cognitive capacity can be voluntarily allocated and that allocation may be dependent on task priority (Tombu and Jolicœur 2003; Wickens 2002). This would mean participants strategically allocated resources to predictable tasks because they were most likely to be accomplished. This interpretation is valid for Experiment 3 because predictability referred to both tasks together and could not be disentangled.

Regarding limitations of our study, theorizing should be addressed first. The interpretations of our results are based on a hypothetical basic premise, namely that resources exist and that resource allocation policy can explain dual-task limitations. As Hommel (2020) recently emphasized, this assumption can neither be falsified nor be replaced by a mechanistic model so far. With this study, however, we did not aim at establishing a mechanism, yet we are aware of the theoretical discourse of the research field. Second, some technical limitations should be mentioned. For example, we interpreted the impact of sequenced tone structures as overall faster RTs, because participants were instructed to press the pedal after hearing the target sound and therefore only responses given after onset were taken into consideration. Even though there was no rhythm and we varied the inter-stimulus intervals, it is possible that participants learned the sequence so well that they gave “anticipatory responses.” Because the tracking software did not capture early responses, any pedal presses ahead of sound onset counted as very late responses to distractor sounds. Hence late responses to distractor sounds in sequenced trials might actually be very early responses to target sounds and thus neither errors nor anticipatory responses could be interpreted with certainty. Future uses of the paradigm should carefully consider three aspects in order to allow more reliable error analyses: varying trial lengths, using different amounts of distractor and target sounds in every trial, and varying inter-stimulus intervals in order to allow for instance d-prime or similar error analyses.

The unique contributions of this study are that it strengthens empirical evidence for the beneficial impact of predictability on performance in general and for the perceptual, cognitive, and motor system’s ability to use covariations in the environment. The implementation of a continuous task and thereby the temporal variable velocity were an important methodological extension to classic tracking/DT studies. Velocities allowed us to examine resource allocation at the moment of interference because they demonstrate changes in tracking behavior during secondary-task processing. This is not possible only with RMSE, which is the standard measure in DT research. Another methodological extension was contrasting ST and DT performance for both tasks instead of only reporting DT costs. This was important to understand resource allocation. Experiment 1 also contributes an innovative redesign of Wulf and Schmidt’s paradigm (1997). Past research has mainly manipulated the middle segment to examine motor learning and its impact on dual tasking, but the implementation of visual information allowed us to examine tracking behavior with online information in a fully unpredictable task environment.

The study also offers practical implications and may guide practitioners who design work spaces or training interventions. First, the workload humans face at work often involves continuous processing and parallel handling of multiple tasks. Our results suggest that, where possible in working spaces, either one task should be made predictable or the environment should allow for tasks’ covariation in space or time. For instance it is possible that an air traffic controller can more efficiently attend to radar control and flight progress strips together, because those two tasks are related in time. Ideally, warning signals help to prepare responses in the more complex task to coordinate tasks more effectively. Second, results from the continuous tracking task may generalize to more complex tasks like driving. Future applied studies should investigate task integration in driving to test the role of predictability. For example, manipulating the temporal positioning of braking signs to effectively maintain steering control might ultimately improve safety in driving. Discussions on using smart phones, voice control, navigation systems, and new technology in (semi-)autonomous driving make such investigations societally relevant. In a related study (Broeker et al. 2020b), participants’ tracking accuracy was compared with performance in a driving simulator and showed that visual predictability has an impact on dual-task driving performance. This is a first step toward generalizing the present results to more applied settings. Third, the finding that task integration improves continuous dual tasks could be relevant for clinical settings and training programs. For instance, if practitioners used co-varying dual tasks such as counting while walking rather than independent dual tasks, performance might improve due to reduced demand for resources and additional risks like falling could be avoided. This would be a promising avenue for further applied research.

## Data Availability

The datasets used and/or analyzed during the current study are available from the corresponding author upon reasonable request.

## Footnotes

1 In a pilot analysis, we compared 200 ms, 400 ms, and 600 ms before onset. We hypothesized that if participants had a constant baseline velocity, these intervals would not differ from each other, and this was indeed the case. Therefore, we used only 200 ms before as the baseline velocity before stimulus onset.

2 This effect is robust given that there was also no effect of predictability on RTs in a replication study with another 28 participants, *F*(2, 54) = 2.20, *p* = .120, η^2^ = .075. In this replication study, only dual-task trials were tested and no dual-task costs were examined, which is why the study is not presented in full here.
